# Pitch Matching in Cochlear Implant Users With Single-Sided Deafness: Effects of Electrode Position and Acoustic Stimulus Type

**DOI:** 10.3389/fnins.2019.01119

**Published:** 2019-11-01

**Authors:** Youssef Adel, Sharon Nagel, Tobias Weissgerber, Uwe Baumann, Olivier Macherey

**Affiliations:** ^1^Audiological Acoustics, Department of Otorhinolaryngology, University Hospital Frankfurt, Frankfurt, Germany; ^2^Aix-Marseille University, CNRS, Centrale Marseille, LMA, Marseille, France

**Keywords:** cochlear implant, pitch perception, single-sided deafness, simulation, pulse-spreading harmonic complex, binary search procedure, non-sensory bias

## Abstract

Previous studies in patients with single-sided deafness (SSD) have reported results of pitch comparisons between electric stimulation of their cochlear implant (CI) and acoustic stimulation presented to their near-normal hearing contralateral ear. These comparisons typically used sinusoids, although the percept elicited by electric stimulation may be closer to a wideband stimulus. Furthermore, it has been shown that pitch comparisons between sounds with different timbres is a difficult task and subjected to various types of range biases. The present study aims to introduce a method to minimize non-sensory biases, and to investigate the effect of different acoustic stimulus types on the frequency and variability of the electric-acoustic pitch matches. Pitch matches were collected from 13 CI users with SSD using the binary search procedure. Electric stimulation was presented at either an apical or a middle electrode position, at a rate of 800 pps. Acoustic stimulus types were sinusoids (SINE), 1/3-octave wide narrow bands of Gaussian noises (NBN), or 1/3-octave wide pulse spreading harmonic complexes (PSHC). On the one hand, NBN and PSHC are presumed to better mimic the spread of excitation produced by a single-electrode stimulation than SINE. On the other hand, SINE and PSHC contain less inherent fluctuations than NBN and may therefore provide a temporal pattern closer to that produced by a constant-amplitude electric pulse train. Analysis of mean pitch match variance showed no differences between stimulus types. However, mean pitch matches showed effects of electrode position and stimulus type, with the middle electrode always matched to a higher frequency than the apical one (*p* < 0.001), and significantly higher across-subject pitch matches for PSHC compared with SINE (*p* = 0.017). Mean pitch matches for all stimulus types were better predicted by place-dependent characteristic frequencies (CFs) based on an organ of Corti map compared with a spiral ganglion map. CF predictions were closest to pitch matches with SINE for the apical electrode position, and conversely with NBN or PSHC for the middle electrode position. These results provide evidence that the choice of acoustic stimulus type can have a significant effect on electric-acoustic pitch matching.

## Introduction

Over the last two decades, the population of hearing-impaired people undergoing cochlear implantation has greatly evolved. While this treatment originally targeted patients with bilateral profound deafness, there are now increasingly more cochlear implant (CI) users with significant residual acoustic hearing in their ipsilateral or, more frequently, contralateral ear. Although this residual hearing is usually restricted to low frequencies, there exists a population of CI users with single-sided deafness (SSD) and normal or near-normal hearing (nNH) in their contralateral ear (Van Zon et al., 2015; Zeitler and Dorman, 2019). In order to enable fusion across the ears of these patients, it may be necessary to deliver to each electrode the frequency information that corresponds to its intracochlear location (Oxenham et al., 2004; Deeks et al., 2013), so that auditory nerve fibers with the same characteristic frequencies (CFs) receive the same information across ears. One way to achieve this is to perform electric-acoustic pitch matching experiments where subjects compare the pitch of a CI electrode with that evoked by acoustic stimuli differing in their spectral content. Previous pitch matching studies have shown that such measurements are difficult to conduct and usually produce very variable data (Carlyon et al., 2010; Goupell et al., 2019). This variability may have several causes, including methodological limitations as well as the choice of the acoustic stimulus type.

### Methods of Electric-Acoustic Pitch Matching

A wide range of methods have been used in the literature to compare the pitches of electric and acoustic stimuli. These include magnitude estimation (Vermeire et al., 2008; Plant et al., 2014), the method of constant stimuli (Boex et al., 2006; Reiss et al., 2007, 2015; Goupell et al., 2019), the method of adjustment (Green et al., 2012; Rader et al., 2016; Maarefvand et al., 2017; Tan et al., 2017) and various kinds of adaptive forced-choice procedures (Reiss et al., 2007; Schatzer et al., 2014; Vermeire et al., 2015; Peters et al., 2016). Carlyon et al. (2010) tested several of these methods and showed they could all be potentially contaminated by different kinds of non-sensory biases. Both magnitude estimation and the method of constant stimuli require the experimenter to predefine a fixed number of acoustic stimuli with which the electrical stimulus will be compared. Carlyon et al. (2010) showed that the choice of this acoustic frequency range could have a large influence on the results. For example, in one subject, the same electrode could be matched to frequencies separated by more than two thirds of an octave for two different acoustic ranges, suggesting that the subjects were not performing real pitch comparisons but rather, and perhaps unconsciously, were basing their judgments on the frequency of the acoustic stimuli only: when the acoustic frequency was high relative to the range, they decided to judge it as “higher” than the electric stimulus, whereas when it was low relative to the range, they judged it as “lower” than the electric stimulus (see also Goupell et al., 2019). This range bias may be very problematic because the range of acoustic frequencies is usually dictated either by the amount of residual hearing of the subjects or by *a priori* estimation of the electric pitch by the experimenter, inferred from radiological findings or from preliminary pitch matches obtained *before* the experiment. An alternative to these procedures that use a fixed range of acoustic frequencies is to perform adjustment or adaptive tasks where the acoustic frequency presented on a given trial depends on the subject’s response to the preceding trial. However, for these tasks, it has been shown that the pitch match can sometimes be strongly correlated with the starting frequency of the procedure, again suggesting that the subjects may not perform pitch comparisons but rather give responses converging near the acoustic stimulus they heard first (Carlyon et al., 2010; Schatzer et al., 2014). Another limitation of adjustment or adaptive tasks is that the pitch of the acoustic stimulus may not vary a lot between consecutive trials, which could distract the subjects from the task itself, especially if the stimuli vary across other dimensions (e.g., loudness or timbre), which may be more salient than the pitch dimension.

The first aim of the present study is to introduce a pitch matching method inspired from the midpoint comparison procedure (Long et al., 2005) and the binary search algorithm used in computer science. The method is relatively time-efficient and attempts to minimize the effects of non-sensory biases: it does not require *a priori* assumption on the frequency range that the electrode should be matched to and it presents acoustic stimuli whose frequency can vary considerably from trial to trial.

### Choice of Acoustic Stimulus Type

Another important concern in electric-acoustic pitch matching experiments is the choice of acoustic stimulus type. While most previous studies have used sinusoids, there is evidence that the percept evoked by electric stimulation via a CI may be very different than that of a pure tone (Lazard et al., 2012). The resulting timbre differences may make pitch comparisons difficult to perform and, therefore, even more prone to non-sensory biases (Carlyon et al., 2010). To our knowledge, only three studies have used stimuli different than sinusoids; Carlyon et al. (2010) used low-rate (12 pps) electric pulse trains and matched them to bandpass-filtered acoustic pulse trains at the same rate. The advantage was that the percepts elicited by these two types of stimuli would be qualitatively similar. It is, however, unclear whether the results of these matches can be extrapolated to higher rates and lower current levels, which are typically used in clinical processors (approximately 1,000 pps). This is because different rates and/or current levels may induce shifts in the spread of excitation and thus influence the “place” pitch percept (e.g., Arnoldner et al., 2006). Green et al. (2012) tested a group of subjects with residual low-frequency hearing and measured pitch matches for different acoustic stimuli, including sinusoids, noise bands and low-rate acoustic pulse trains. Their results were very variable, but they found in one subject that the matched frequency was significantly different when using acoustic pulse trains than for sinusoids and noise bands. Maarefvand et al. (2017) have used sinusoids as well as harmonic complex tones and found that the pitch-matched frequency for each electrode was always higher when using a pure tone than the harmonic complex tone. Thus far, there is only limited evidence that the choice of acoustic stimulus type can have an effect on the electric-acoustic pitch matching results. Recently, pulse-spreading harmonic complexes (PSHCs) have been proposed as an alternative to simulate electric pulse stimulation via a CI (Hilkhuysen and Macherey, 2014). The design of PSHC addresses some of the limitations of sinusoidal or noise carriers commonly used for CI simulations, i.e., that sinusoids cannot simulate the broad spread of excitation produced by an electric current pulse and that noise bands contain intrinsic modulations which are absent in pulse trains with a constant current amplitude. PSHCs are pulsatile broadband stimuli that can simulate the broad spread of excitation and their pulse rate can be adjusted to minimize intrinsic modulations after auditory filtering (Mesnildrey et al., 2016). Furthermore, a recent evaluation in SSD subjects showed that speech processed by a vocoder using PSHC carriers was judged more similar to speech processed by the clinical CI processor than sine- or noise-vocoded speech (Karoui et al., 2019).

**FIGURE 1:**
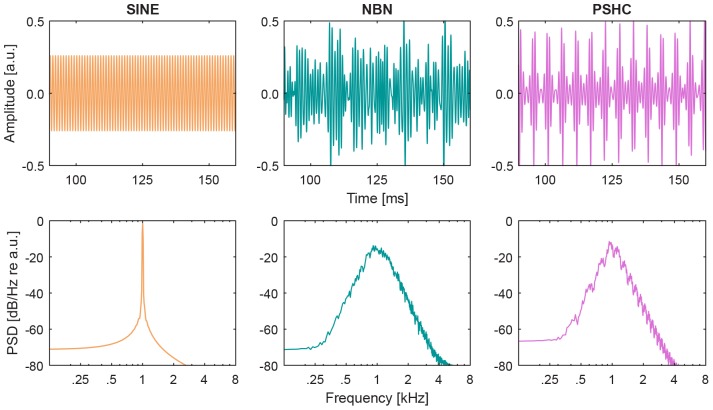
Stimulus waveforms **(upper panels)** and power spectrum densities (PSD, **lower panels**) are shown for each acoustic stimulus type: sinusoid (SINE), narrow-band noise (NBN), and pulse spreading harmonic complex (PSHC), all centered at 1 kHz.

The second aim of the present study is to test the hypothesis that PSHCs can yield less variable pitch matches compared with sinusoids (SINE) and narrow-band noises (NBN, see Figure 1). Pitch was matched for an apical and a middle electrode position (E1 and E6, respectively) and compared with place-dependent CFs predicted by empirical models. Our underlying assumption was that a signal perceptually more similar to the CI should yield less variable electric-acoustic pitch matches.

## Materials and Methods

### Subjects

**FIGURE 2:**
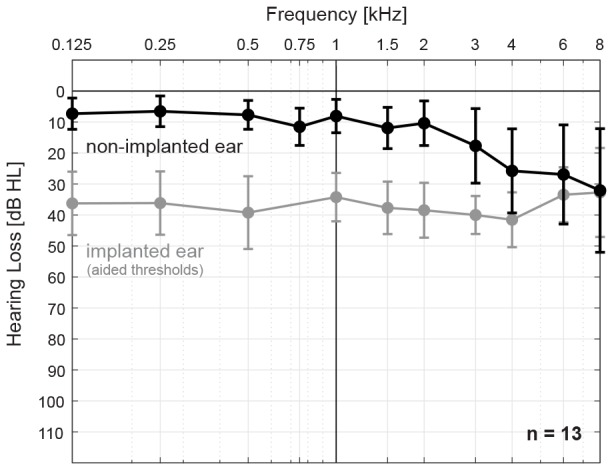
Mean and standard deviation air conduction thresholds for the non-implanted ear, i.e., near-normal hearing (nNH, black) and implanted ear, i.e., cochlear-implant aided thresholds (gray) for all subjects (*n* = 13).

Thirteen subjects (3 female, 10 male) with late-onset SSD and nNH in the contralateral ear participated in the study. Pure-tone air conduction thresholds were less than or equal to 20 dB HL in the frequency range from 125 to 2 kHz for all subjects, and up to 60 dB HL in the frequency range 3–8 kHz. Mean and standard deviation air conduction thresholds for the non-implanted and implanted (i.e., aided thresholds) ear, respectively, are shown in Figure 2. All subjects were experienced CI users (range 11 months – 7 years after implantation) with Concerto or Synchrony devices (MED-EL, Innsbruck, Austria) and either the 28-mm Flex28 electrode array (*n* = 10) or the 31.5-mm FlexSoft electrode array (*n* = 3). Subject demographics are provided in Table 1. Electrode migration occurred in one subject (S13, denoted by ^∗^) 5 years prior to the study and consequently, the two most basal electrodes were turned off. Tinnitus was reported in the implanted ear by 8 subjects (denoted by ^+^), one of whom also reported it in the non-implanted ear (S07). All subjects received financial compensation and reimbursement of their traveling costs. This study was carried out in accordance with the ethical standards of the institutional review board at the Goethe University Frankfurt, which approved the study protocol (IRB approval number 209/13). All subjects gave written informed consent in accordance with the Declaration of Helsinki.

**TABLE 1:** Subject demographics.

**Subject**	**Implanted Ear**	**Age at Implantation [years]**	**Duration of CI use [years]**	**Age at Onset of Hearing Loss [years]**	**Etiology**
S01	R	49	6	45	Sudden hearing loss
S02^+^	L	68	4	58	Sudden hearing loss
S03	R	70	5	66	Sudden hearing loss
S04^+^	L	53	5	40	Progressive hearing loss
S05^+^	R	45	5	43	Sudden hearing loss
S06	R	67	1	59	Toxic otitis
S07^+^	L	47	4	41	Sudden hearing loss
S08^+^	L	62	2	40	Head trauma
S09	R	69	7	65	Sudden hearing loss
S10^+^	L	37	1	36	Meningitis
S11^+^	L	60	3	57	Sudden hearing loss
S12^+^	R	53	<1	51	Sudden hearing loss
S13^∗^	L	68	6	64	Sudden hearing loss

*L, left; R, right. ^∗^Two most basal electrodes were turned off. ^+^Tinnitus was reported in the implanted ear.*

### Stimuli

Electric stimuli were 400-ms biphasic cathodic-first pulse trains presented in monopolar mode. The two phases were symmetric and rectangular, had durations of 45 μs each, and were separated by a 2.1-μs inter-phase gap. All electric pulse trains were presented at a rate of 800 pps.

Acoustic stimuli were either sinusoids (SINE), 1/3-octave wide narrow bands of Gaussian noises (NBN), or 1/3-octave wide pulse spreading harmonic complexes (PSHC; Hilkhuysen and Macherey, 2014). They had a duration of 400 ms and 20-ms raised cosine onset and offset ramps. They were presented at (center) frequencies ranging between 125 and 4 kHz. NBN and PSHC were spectrally limited outside the 1/3-octave passband using 6^th^ order Butterworth low-pass and high-pass filters, i.e., they had 36 dB per octave spectral slopes. PSHCs were presented at center frequency-dependent optimal pulse rates according to Mesnildrey et al. (2016). Figure 1 shows waveform excerpts, i.e., amplitude over time (upper panels), and power spectrum densities (PSD, lower panels) for each stimulus type centered at 1 kHz.

### Procedures

Prior to the experiment, pure-tone air conduction thresholds of the nNH ear were measured and CI electrode impedances of the implanted ear were checked. As described in detail below, the experiment started by determining electric (CI) and acoustic (nNH) loudness profiles to establish electric current and sound pressure levels at comfortable loudness. This was followed by balancing the loudness between the two stimulation modalities. After an enforced break and a short acoustic pitch demonstration, an electric pitch ranking task was conducted to verify that the subjects had no pitch reversals for the relevant electrodes. Thereafter, acoustic-acoustic pitch matching was conducted as a control measure of subjects’ ability to perform pitch comparisons. Finally, electric-acoustic pitch matching procedures were carried out using the binary search procedure.

#### Electric and Acoustic Loudness Profiles

Electric current or sound pressure levels were initially adjusted to most-comfortable loudness (MCL), defined as rating 6 on a 10-point rating scale. Electric loudness profiles were determined by presenting pulse trains to a single CI electrode at a time, while monitoring subjects’ loudness perception on a 10-point rating scale. Current level was increased from 94.5 CU (current units, 1 CU ≈ 1 μA) in steps of 1, 2, or 4 times 9.45 CU from rating 0 (“no percept”) up to rating 7 (“loud but comfortable”), then decreased back in steps of 9.45 CU to the final rating 6 (“most comfortable”). MCL current levels were determined for electrodes E1 (apical) to E8 (basal) in ascending order. Hereafter, electric stimuli were always presented at these MCL levels.

Acoustic loudness profiles were determined for each stimulus type, i.e., SINE, NBN, or PSHC, in random order. Analogous to the electric loudness profile, a given stimulus type was presented while monitoring subjects’ loudness perception on the 10-point rating scale. Initial frequency-dependent sound pressure levels were based on pilot loudness-adjustment tests in NH subjects (*n* = 4, data not shown here) using the same acoustic stimuli and anchored on a 1-kHz pure tone at 65 dB SPL. Sound pressure levels were increased in steps of 1, 2, or 4 dB up to rating 7 and then decreased back in steps of 1 dB to the final rating 6. MCL sound pressure levels were determined for a given stimulus type starting at a (center) frequency of 1 kHz up to 4 kHz in half-octave steps, and then from 1 kHz down to 125 Hz in half-octave steps.

#### Electric-Acoustic Loudness Balancing

After having determined electric and acoustic loudness profiles, an adjustment paradigm adopted from Macherey and Carlyon (2010) was used to balance loudness between the two stimulation modalities. Each loudness-balancing trial consisted of an electric pulse train presented to the CI ear followed by an acoustic stimulus presented to the nNH ear after a 400-ms inter-stimulus gap. The electric pulse train was the reference and its level was fixed at MCL throughout a given adjustment task. The acoustic stimulus had an initial level of MCL ± 6 dB and was adjustable in steps of 0, 1, 2, or 4 dB using a graphical user interface provided to the subjects. They were asked to balance the loudness of the acoustic stimulus to that of the electric stimulus and were encouraged to make over- and undershoots before deciding on the final level. A minimum of 10 level adjustments was enforced before subjects could indicate that loudness was balanced and terminate a given adjustment task.

This procedure was carried out once for each combination of electrode (E3 or E4) and acoustic stimulus type (SINE, NBN, or PSHC) presented at different initial levels (MCL ± 6 dB), i.e., 12 possible combinations, in random order. Each acoustic stimulus was presented at a (center) frequency selected randomly without replacement from the set of frequencies ranging between 125 and 4 kHz in half-octave steps. Loudness was finally balanced for each acoustic stimulus by applying the mean adjustment of two electrodes (E3 and E4) and two different initial levels (MCL ± 6 dB), i.e., from 4 conditions, to the respective acoustic loudness profile. Each profile was then linearly interpolated by a factor of 12 to obtain a quarter-tone (i.e., 50 cents) frequency spacing. Hereafter, acoustic stimuli were always presented at these loudness-balanced MCL levels.

Note that the electrodes used for electric-acoustic loudness balancing (E3 and E4) were different than those used for electric-acoustic pitch matching (E1 and E6, see below) in order to prevent having subjects compare in loudness the same electric stimuli they would later compare in pitch, thereby avoiding such loudness comparisons from providing an additional source of bias (McDermott et al., 2008).

#### Acoustic Pitch Demonstration

After an enforced break at the end of the loudness balancing tasks, a short acoustic pitch demonstration was presented to accustom the subjects to the new tasks concerning pitch perception. Each demonstration trial consisted of two acoustic stimuli presented to the nNH ear, separated by a 400-ms inter-stimulus gap. Each combination of acoustic stimulus (SINE, NBN, or PSHC) and frequency order (ascending or descending) was presented three times, in random order. For each trial, a pair of (center) frequencies was selected randomly without replacement from the set of frequencies ranging between 125 and 4 kHz in half-octave steps. Each stimulus playback was visually cued and feedback was provided to indicate which stimulus was higher in pitch. Subjects were asked to listen and compare their judgment to the provided feedback.

#### Electric Pitch Ranking

In order to verify that the subjects had no pitch reversals for the relevant electrodes in the CI ear, the midpoint comparison procedure was used to rank electrodes according to their pitch. This procedure was adopted from Long et al. (2005), who originally developed it to optimize the fitting of auditory brainstem implants, and its implementation in CI users has been previously described in Macherey and Carlyon (2010). The procedure starts by randomly selecting a pair of electrodes without replacement from the set of electrodes to be ranked. Electric pulse trains were presented to each electrode, separated by a 400-ms inter-stimulus gap. The subjects’ task was to indicate which electrode was higher in pitch, with the order of presentation randomized between trials. The procedure continues by randomly selecting additional electrodes in order to gradually rank the set of electrodes according to their pitch. To briefly illustrate this, assume that at one point of the procedure the provisional ranking of the electrodes was [E1, E3, E2, E7, E8]. The randomly selected electrode to be ranked next is E4, and is first compared to the middle-ranked electrode E2. Given an electrode pair, in this case E4 and E2, each trial consisted of an electric pulse train presented to one electrode and then to the other, with the order of electrodes randomized between trials. Each stimulus playback was visually cued without feedback. If the subject ranked one electrode higher two times in a row, then it was defined as their response. Otherwise, a third trial was presented and its result defined as their response. This best-of-three format was added to the original procedure of Long et al. (2005) to minimize confounding factors, such as lack of concentration (Levitt and Rabiner, 1967). In this illustration, if E4 was finally ranked higher in pitch than E2, then the list would be bisected and E4 compared to the consequently middle-ranked stimulus E7. If it was thereupon ranked lower in pitch than E7, then the probed electrode E4 would be added to a new provisional ranking between E2 and E7, i.e., [E1, E3, E2, E4, E7, E8]. Subsequently, the next electrode to be ranked would be randomly selected and the procedure repeated until all electrodes are ranked.

The procedure was carried out 3 times for electrodes E1 to E8 with the requirement of no pitch reversals for electrode pairs [E1, E4], [E3, E6], and [E1, E6] in at least 2 out of the 3 repetitions. These pairs were later used for catch trials and electric-acoustic pitch matching.

#### Acoustic-Acoustic Pitch Matching

**FIGURE 3:**
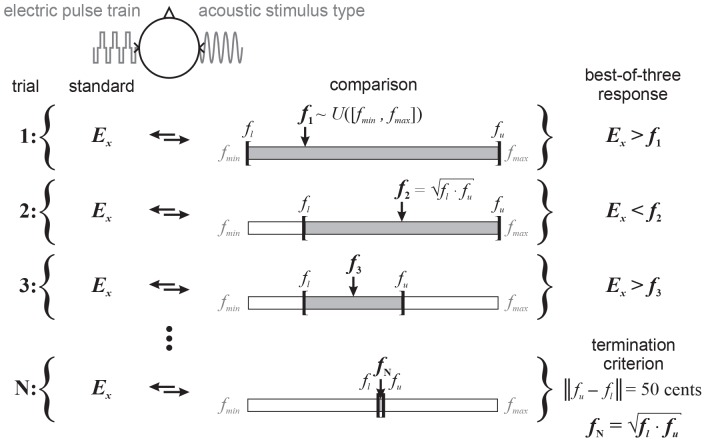
Illustration of the *binary search procedure*: each trial consists of a standard electric pulse train fixed in electrode position (*E*_*x*_) throughout a given pitch matching run (trials 1 to N), and an acoustic stimulus type. Subjects are asked to indicate whether the standard or comparison is higher in pitch, with the order of presentation randomized between trials. The (center) frequency (*f*_*x*_,*x* ∈ {1,2,3,…,N}) of the acoustic stimulus is adaptively changed according to their response, which is evaluated in a best-of-three format for each trial. The starting frequency is randomly drawn from a uniform distribution, i.e., *f*_1_∼*U*([*f*_*min*_,*f*_*max*_]). The pitch matching range [*f*_*l*_,*f*_*u*_] initially has the same lower and upper boundaries. In this illustration, the electric stimulus is perceived higher in pitch than the acoustic stimulus at the starting frequency (trial 1), i.e., *E*_*x*_ > *f*_1_. Consequently, the lower boundary is set to that frequency and the next frequency (trial 2) set to the geometric mean of the current lower and upper boundaries, i.e., $f_{2}=\sqrt{f_{l}\cdot f_{u}}$. The electric stimulus is then perceived lower in pitch than the acoustic stimulus, i.e., *E*_*x*_ < *f*_2_, and the upper boundary is set to that frequency. The next frequency (trial 3) is again set to the geometric mean of the current boundaries. This iterative process is terminated (trial N) when the difference between lower and upper boundaries is a quarter-tone (i.e., 50 cents). The final pitch match *f*_*N*_ is defined as the geometric mean of the final boundaries.

As a control measure of subjects’ ability to perform the final procedure, they were asked to match the pitch between two acoustic stimuli presented to the nNH ear, separated by a 400-ms inter-stimulus gap. Each trial consisted of a standard stimulus fixed in (center) frequency throughout a given pitch matching run, and a comparison stimulus whose frequency was adaptively changed according to the binary search procedure described below (see Figure 3). Each stimulus playback was visually cued without feedback. This procedure was carried out 3 times for each combination of standard frequency (250 Hz or 1 kHz) and acoustic stimulus (SINE, NBN, or PSHC), in random order.

Between-subject pitch matching variability was compared with data acquired from pilot pitch matching tests in NH subjects (*n* = 10, data not shown here) using the same procedure and acoustic stimuli (NBN and PSHC) to test the effect of spectral slope on matching accuracy, with the standard fixed at 36 dB per octave and the comparison at 24, 36, or 48 dB per octave. Key differences in the NH experiment were that (i) standard and comparison stimuli were presented to contralateral ears, with the order randomized between subjects, (ii) the pitch matching range was up to 8 kHz compared with 4 kHz for SSD subjects, and (iii) NH subjects were also tested using the standard frequency of 4 kHz.

#### Electric-Acoustic Pitch Matching

The main and final task was to compare the pitch of an electric pulse train presented to the CI ear to that of an acoustic stimulus presented to the nNH ear as illustrated in Figure 3. Each pitch matching trial consisted of a standard electric stimulus fixed in electrode position (*E*_*x*_) throughout a given pitch matching run (trials 1 to N), and an acoustic stimulus type, separated by a 400-ms inter-stimulus gap. The subjects’ task was to indicate whether the standard or comparison was higher in pitch, with the order of presentation randomized between trials. Each stimulus playback was visually cued without feedback. The (center) frequency (*f*_*x*_,*x* ∈ {1,2,3,…,N}) of the acoustic stimulus was adaptively changed according to the *binary search procedure*: The starting frequency was randomly drawn from a uniform distribution ranging from 125 Hz (*f*_*min*_) to 4 kHz (*f*_*max*_), i.e., *f*_1_∼*U*([*f*_*min*_,*f*_*max*_]). And the pitch matching range [*f*_*l*_,*f*_*u*_] initially had the same lower and upper boundaries, i.e., 125 Hz and 4 kHz, respectively. Subjects’ response was evaluated in the best-of-three format as described above. In the illustration shown in Figure 3, the electric stimulus was perceived higher in pitch than the acoustic stimulus at the starting frequency (trial 1), i.e., *E*_*x*_ > *f*_1_. Consequently, the lower boundary was set to that frequency and the next frequency (trial 2) set to the geometric mean of the current lower and upper boundaries, i.e., $f_{2}=\sqrt{f_{l}\cdot f_{u}}$. The electric stimulus was then perceived lower in pitch than the acoustic stimulus, i.e., *E*_*x*_ < *f*_2_. In this case, the upper boundary was set to that frequency and the next frequency (trial 3) was again set to the geometric mean of the current boundaries. This iterative process was terminated (trial N) when the difference between the lower and upper boundaries was a quarter-tone (i.e., 50 cents). The pitch match *f*_*N*_ was then defined as the geometric mean of the final boundaries.

This procedure was repeated five times for each combination of electrode (E1 or E6) and acoustic stimulus type (SINE, NBN, or PSHC) in random order. A short break was enforced in the middle of the entire procedure to minimize the effect of fatigue. A given pitch matching run could be interrupted by two types of catch trials, electric or acoustic, each with 15% probability of occurrence. Electric catch trials randomly selected an electrode pair, [E1, E4] or [E3, E6], and presented an electric pulse train to each electrode in random order. Acoustic catch trials randomly selected a pair of (center) frequencies without replacement from the set of frequencies ranging between 125 Hz and 4 kHz in half-octave steps. The stimulus type tested in the current pitch matching run was presented at each frequency in random order. The subjects’ task was still to indicate which stimulus was higher in pitch. Each stimulus playback was visually cued with feedback. If the response was correct, then the pitch matching run was immediately resumed. Otherwise, the catch trial was repeated twice. These catch trials aimed to impel subjects to focus on the pitch dimension by restricting these trials to a single modality, and to give them positive reinforcement on relatively easy trials.

### Materials

Electric stimuli were directly transmitted to the CI using the Research Interface Box II (RIB, University of Innsbruck, Innsbruck, Austria). Acoustic stimuli were presented using a D/A converter and amplifier (24 bit, 48 kHz sampling rate, RME Fireface UC, Haimhausen, Germany), and audiometric headphones (Sennheiser HDA 200, Wedemark, Germany). All experimental procedures and graphical user interfaces were programmed in MATLAB (MathWorks, Natick, MA, United States) using the RIB library and Psychophysics Toolbox extensions (Brainard, 1997) to respectively drive the electric and acoustic stimulation hardware.

### Analysis

Data are generally presented as geometric mean, or plotted as boxplots with mean values included as circles. Within-subject comparisons were calculated using repeated-measures analyses of variance (ANOVA) and Tukey’s LSD post-hoc test. Linear correlation between measures was tested using Pearson’s *r*. A *p-*value of less than 0.05 was considered statistically significant. Statistical analysis was performed using SPSS Statistics (IBM Corporation, Endicott, NY, United States).

In addition to analyzing electrode positions using their order on the electrode carrier, angles of insertion were estimated using postoperative X-ray images acquired with the modified Stenvers’ projection (cochlear view, Xu et al., 2000), which has been described and illustrated in previous studies (Verbist et al., 2010; Rader et al., 2016).

## Results

### Control Procedures

Electric pitch ranking using the midpoint comparison procedure was conducted to verify that the subjects had no pitch reversals for the relevant electrode pairs, which were [E1, E4] and [E3, E6] for the catch trials, and [E1, E6] for the electric-acoustic pitch matching. Only 2 subjects (S09 and S11) had pitch reversals for the pair [E3, E6] and 1 subject (S09) for the pair [E1, E6], each of which only occurred in 1 out of the 3 repetitions. Median rank differences for all subjects were 3 ranks between [E1, E4] (range 1 – 5), 3 ranks between [E3, E6] (range 2 – 5), and 5 ranks between [E1, E6] (range 4 – 6). If evaluated using the best-of-three format, 4 subjects (31%) had a “perfect” ranking, i.e., they ranked the electrodes in ascending order from E1 to E8 without any pitch reversals. If divided into the 4 apical (E1 to E4) and 4 basal (E5 to E8) electrodes, 5 subjects (38%) had perfect ranking for the apical electrodes and 8 subjects (62%) had perfect ranking for the basal electrodes.

Acoustic-acoustic pitch matching was conducted as a control measure of subjects’ ability to perform pitch comparisons prior to collecting electric-acoustic pitch matching data. Pitch match distributions were comparable between conditions, which was confirmed using a two-way repeated-measures ANOVA with the factors standard frequency (250 Hz or 1 kHz) and stimulus type (SINE, NBN, or PSHC), which showed neither within-subject effects nor interaction effects. Mean absolute deviation from either standard (250 Hz or 1 kHz) was 2 semitones or lower for NBN and PSHC, which was comparable with the NH results. SINE showed the highest variance and had a mean absolute deviation of over 3 semitones from the 250-Hz standard, which could be due to octave confusions (Lockhead and Byrd, 1981). For the 4-kHz standard tested in NH subjects, mean absolute deviation was 10 semitones (minor seventh) for NBN and 8 semitones (minor sixth) for PSHC. This finding showed that pitch matching accuracy for NBN and PSHC substantially decreased at high frequencies and was thus not included in the testing of SSD subjects.

Electric and acoustic catch trials were carried out during the electric-acoustic pitch matching procedure to verify and if necessary impel subjects to focus on pitch. Mean percent correct responses were 93% for electric catch trials, 97% for SINE, 94% for NBN, and 97% for PSHC. These relatively high scores strongly suggest that the positive reinforcement of the subjects to focus on the pitch dimension was achieved.

Finally, no systematic correlations were found between starting frequency and final pitch matches, which suggests that the binary search procedure was not contaminated by this limitation previously observed for other adaptive methods (Carlyon et al., 2010). A possible explanation is that the change in frequency during the first trials is very large due to the fundamental approach of the binary search algorithm, which progressively bisects the frequency range. Therefore, it would require the subject an active effort to return to the starting frequency during the procedure in case they were not in fact comparing each matching trial independently.

### Pitch Match Mean and Variance

**FIGURE 4:**
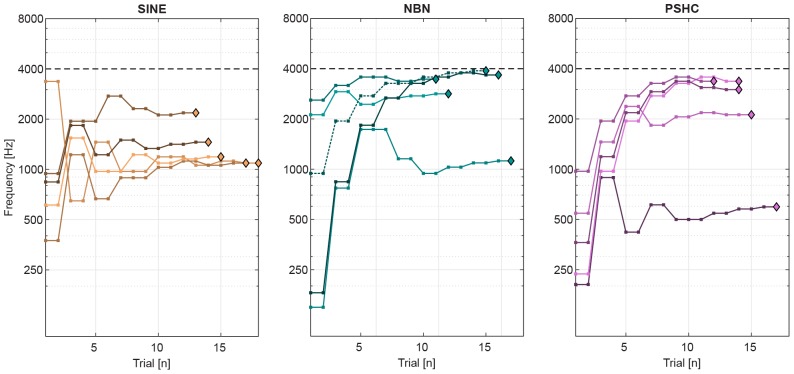
Example of pitch matching runs for each stimulus type (SINE, NBN, or PSHC), for a given subject (S11) and electrode position (E6). The dashed horizontal gridline shows the maximum frequency at 4 kHz. For each trial, comparison (center) frequency is shown as squares. And the final pitch matches are shown as diamonds. In one pitch matching run of NBN (dashed line), the subject appears to have perceived the pitch very close to (or possibly above) the maximum frequency, but was hindered by the procedure’s parameters.

Figure 4 shows an example of pitch matching runs for each stimulus type (SINE, NBN, or PSHC), for a given subject (S11) and electrode position (E6). The dashed horizontal gridline shows the maximum frequency at 4 kHz (*f*_*max*_, see Figure 3). In one pitch matching run of NBN (dashed line), the subject appears to have perceived the pitch very close to (or possibly above) the maximum frequency, but was hindered by the procedure’s parameters. This cap occurred in up to 2 pitch matching runs in 4 conditions using NBN, and in 3 conditions using PSHC (see Table 2, denoted by ^∗^), which were all at E6 except for once at E1. In order to take these capped trials into account, maximum-likelihood estimations (MLE) of pitch match mean and variance were calculated; for each subject and each condition, the likelihood of obtaining the collected data was computed for a wide range of means and standard deviations. The probability of obtaining a data point below 4 kHz was based on the normal probability density function, while the probability of obtaining a data point above 4 kHz, i.e., a capped trial, was based on the upper tail probability of the normal distribution. Hereafter, all pitch match means and variances are MLE values unless otherwise stated. In some cases, all pitch matching runs were capped and the respective condition was thus excluded from the analysis (see Table 2).

**TABLE 2:** Individual MLE pitch match [Hz].

**Subject**	**E1**			**E6**		
		
	**SINE**	**NBN**	**PSHC**	**SINE**	**NBN**	**PSHC**
S01	240.5	408.3	417.9	1233.2	−	3639.4
S02	200.0	257.6	246.1	399.1	935.5	2349.8^∗^
S03	331.9	302.7	427.6	1124.7	1663.5	1663.5
S04	647.2	2046.6	1702.3	935.5	3027.1^∗^	2296.3
S05	339.6	538.3	709.6	853.2	4688.5^∗^	2958.2^∗^
S06	363.9	155.2	186.7	502.4	709.6	1415.9
S07	339.6	576.8	427.6	1049.6	1517.2	1205.1
S08	200.0	381.1	632.5	1824.0	−	−
S09	324.4	3724.2^∗^	1049.6	381.1	−	1910.0
S10	257.6	1482.6	853.2	760.4	−	3243.6^∗^
S11	195.4	276.1	347.6	1352.2	2958.2^∗^	2094.3
S12	662.3	576.8	490.9	2296.3	1663.5	1625.7
S13	246.1	295.8	381.1	1702.3	1074.1	1866.5
*Mean*	308.7	537.4	509.5	967.5	1711.0	2078.2

*^∗^, up to 2 matches were capped by maximum frequency at 4 kHz; –, excluded conditions because all matches were capped; MLE, maximum-likelihood estimated.*

**FIGURE 5:**
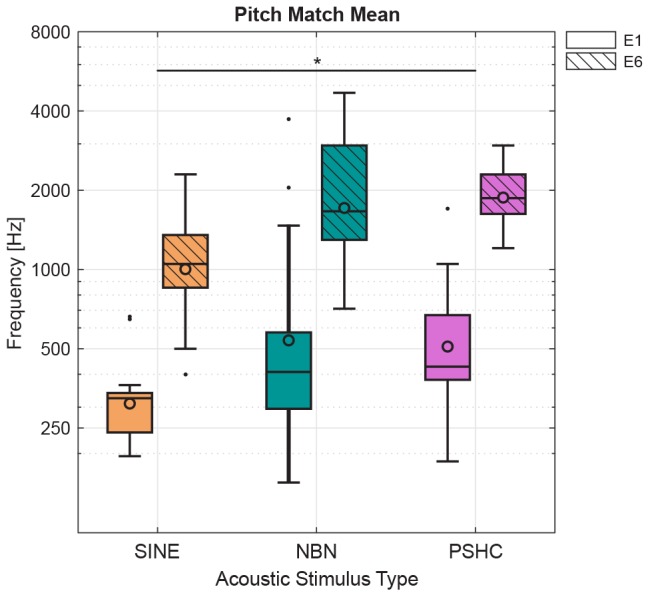
Pitch match means for each stimulus type (SINE, NBN, or PSHC) and for each electrode position (E1 or E6) as boxplots with grand geometric means indicated as circles. Two-way repeated-measures ANOVA showed a significant effect of electrode position [*F*(1,8) = 74.1, *p* < 0.001] and acoustic stimulus type [*F*(2,16) = 5.50, *p* = 0.015]. Pairwise comparisons showed significant differences between electrode positions E1 and E6 (*p* < 0.001, not shown here) and between stimulus types SINE and PSHC (^∗^*p* = 0.017), whereas the difference between SINE and NBN was marginally not significant (*p* = 0.07).

Figure 5 shows pitch match means for each stimulus type (SINE, NBN, or PSHC) and for each electrode position (E1 or E6) as boxplots with grand geometric means indicated as circles. Table 2 shows grand geometric means for all conditions. A two-way repeated-measures ANOVA was performed with the factors electrode position and stimulus type (*n* = 9 due to exclusions). Mauchly’s test indicated that the assumption of sphericity was not violated for any of the model effects. Both within-subject effects were significant, with *F*(1,8) = 74.1, *p* < 0.001 for the electrode position effect, and *F*(2,16) = 5.50, *p* = 0.015 for the stimulus type effect. No interaction effect was observed. Pairwise comparisons showed significant differences between electrode positions E1 and E6 (*p* < 0.001) and between stimulus types SINE and PSHC (*p* = 0.017), whereas the difference between SINE and NBN was marginally not significant (*p* = 0.07). The two-way repeated-measures ANOVA was repeated with ‘actual’ instead of MLE values and yielded equivalent results.

**FIGURE 6:**
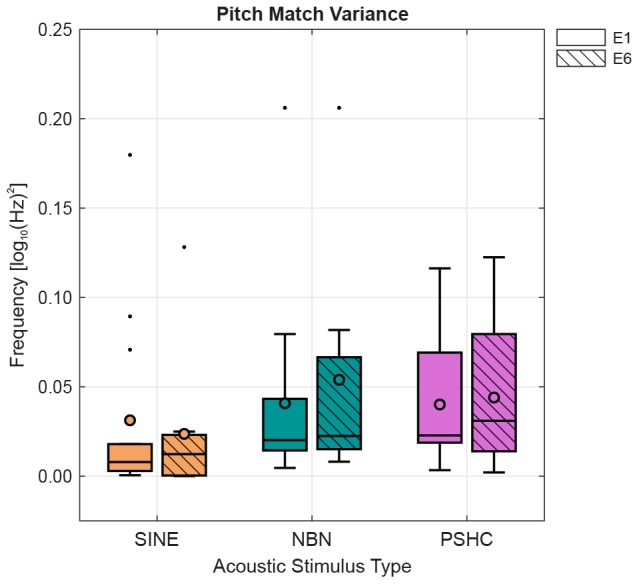
Pitch match variances for each stimulus type (SINE, NBN, or PSHC) and for each electrode position (E1 or E6) as boxplots with mean variances indicated as circles. Two-way repeated-measures ANOVA showed neither within-subject effects nor interaction effects.

Figure 6 shows pitch match variances for each stimulus type and electrode position as boxplots with mean variances indicated as circles. SINE generally showed lower mean or median variances. And while SINE and NBN showed a few outliers, the distributions were generally comparable between electrode positions and stimulus types. This was confirmed by a two-way repeated-measures ANOVA showing neither within-subject effects nor interaction effects. Consequently, we were not able to reject the null hypothesis regarding the effect of stimulus type on the variability of pitch matches.

### Pitch Match as a Function of Angle of Insertion

**FIGURE 7:**
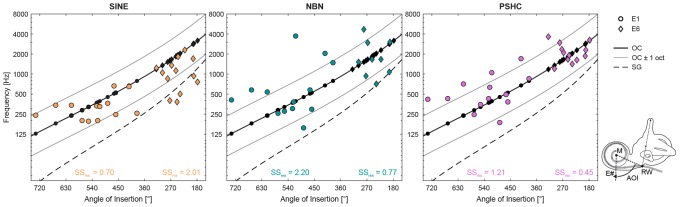
Pitch match means for each stimulus type (SINE, NBN, or PSHC) and for each electrode position (E1 as circles, and E6 as diamonds) as a function of angle of insertion (AOI) estimated using the modified Stenvers’ projection (Verbist et al., 2010). The schematic of a left cochlea shows how the AOI was measured for a given electrode (E#) by clockwise rotation at the geometric zero reference, which was defined as the line between the crossing point of the electrode array (gray) with the round window (RW), and the modiolus (M). The organ of Corti (OC) frequency map ± 1 octave (solid black and gray curves, respectively) and the spiral ganglion (SG) frequency map (dashed black curve) are repeated in each panel. Predicted characteristic frequencies according to the OC map (black filled circles or diamonds) are shown in each panel along the OC map’s curve. Residual sum of squares [SS_res_, expressed in log_10_(Hz)] for the OC map is shown for each combination of acoustic stimulus type and electrode position.

Figure 7 shows pitch match means for each stimulus type (SINE, NBN, or PSHC) and for each electrode position (E1 or E6) as a function of angle of insertion, which was estimated from postoperative X-ray images. Based on a histological study (Stakhovskaya et al., 2007), angles of insertion were transformed to percentage distances (or lengths) of the organ of Corti (OC) or the spiral ganglion (SG). These were then mapped to place-dependent characteristic frequencies (CFs) according to the empirically modeled Greenwood function (Greenwood, 1990). The OC frequency map ± 1 octave (solid black and gray curves, respectively) and the SG frequency map (dashed black curve) are repeated in each panel.

Residual sum of squares (SS_res_) was calculated for each combination of stimulus type and electrode position as a measure of deviation of pitch match means from predicted CFs according to the OC or SG map. For the SG map, SINE had generally the smallest deviation for both electrode positions. However, the OC map reduced deviations for all stimulus types and electrode positions by at least a factor of 3 (up to 8) compared with the SG map, except for SINE at E6 with an increase of 47%. This suggests that the OC map was generally a better model for our results, which is consistent with the fact that the implanted Flex28 or FlexSoft electrodes are typically placed homogenously along the lateral wall (Dhanasingh and Jolly, 2017). In Figure 7, SS_res_ expressed in log_10_(Hz) are shown for the OC map, for each condition. For E1, SINE had the smallest deviation, followed by PSHC, and then NBN. And for E6, PSHC had the smallest deviation, followed by NBN, and then SINE, which was generally matched lower than the predicted CFs.

These data suggest an inverse relation between electrode position and the deviation of SINE versus NBN or PSHC, which was largely confirmed by one-way repeated-measures ANOVA with the factor stimulus type for each electrode position: For E1, there was a significant within-subject effect of stimulus type, with *F*(2,24) = 5.8, *p* < 0.01. Pairwise comparisons showed that SINE pitch match means were significantly closer to CFs predicted by the OC map than either NBN or PSHC (*p* = 0.035 and *p* < 0.01, respectively). For E6, within-subject effect of stimulus type was also significant, with *F*(2,16) = 5.4, *p* = 0.016. But contrary to E1, pairwise comparisons for E6 showed that NBN and PSHC were closer to predicted CFs than SINE (*p* = 0.046 and *p* = 0.021, respectively). In both analyses, no significant differences were found between NBN and PSHC. Note, however, that the differences and by extension the inverse relation appear to be larger between SINE and PSHC than between SINE and NBN.

## Discussion

In this study, we minimized the effect of sensory and non-sensory biases on electric-acoustic pitch matches in CI users with SSD by controlling for loudness profiles, balancing between the two modalities, accounting for possible reversals in electric pitch perception, and implementing the binary search procedure to match pitch between electric and acoustic stimuli. While the mean pitch match variance showed no differences between acoustic stimulus types, mean pitch matches showed effects of electrode position and stimulus type, with the middle electrode always matched to a higher frequency than the apical one, and significantly higher across-subject pitch matches for PSHC compared with SINE. Mean pitch matches for all stimulus types were better predicted by CFs according to the OC map than the SG map. CF predictions were closest to pitch matches with SINE for the apical electrode position, and conversely with NBN or PSHC for the middle electrode position. In the following, we consider methodological limitations of the study design and then discuss the observed effects of acoustic stimulus type and electrode position on electric-acoustic pitch matches.

### Binary Search Procedure

**FIGURE 8:**
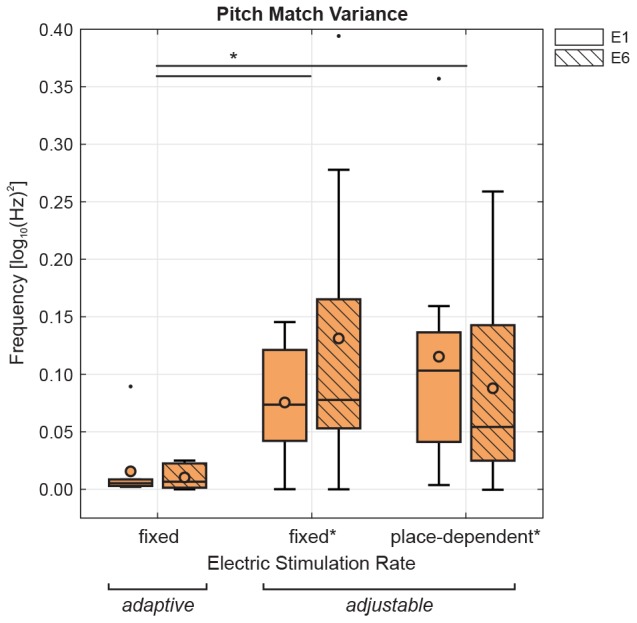
Pitch match variances for each electrode position (E1 or E6) are compared in a subset of subjects previously tested using SINE (*n* = 9, Rader et al., 2016). Pitch matches were collected using either the binary search procedure (adaptive, from the current study) at a fixed rate of 800 pps, or the method of adjustment (adjustable, from the previous study as denoted by ^∗^) at a fixed rate of 800 pps or at the place-dependent rate. Two-way repeated-measures ANOVA showed a significant effect of matching method, with the binary search procedure significantly lower than the method of adjustment at either rate.

Although the binary search procedure has not been directly compared with other pitch matching methods, 9 of the subjects who participated in the present study were also subjects in a previous study by Rader et al. (2016) which used the method of adjustment. The acoustic stimulus type was SINE, and was matched with electric stimuli in two different conditions: pulse trains either had a fixed rate of 800 pps or they had a rate corresponding to predicted CF for the intracochlear electrode location, i.e., place-dependent rates. For each condition, 6 repetitions were obtained for electrodes E1– E6. Figure 8 shows the variance of the pitch matches obtained for E1 and E6 using the binary search procedure (adaptive) at a fixed rate of 800 pps, and for the two adjustment procedures (adjustable) of Rader et al. (2016) at a fixed rate of 800 pps or at the place-dependent rate. While these data were not all collected on the same day and are, therefore, not directly comparable, it is worth noting that the binary search procedure could yield a much smaller variance than the adjustment procedure. This was confirmed by a two-way repeated-measures ANOVA with the factors electrode position (E1 or E6) and pitch matching method (adaptive with fixed rate, adjustable with fixed or place-dependent rate). Results showed no effect of electrode position but a significant effect of method, *F*(2,14) = 7.25, *p* = 0.007, with lower variance of the adaptive method, i.e., binary search procedure, than either adjustable methods. If this observation was confirmed in a direct comparison of pitch matching methods, it could mean that the binary search procedure is easier to perform than an adjustment task. Comparing electric-acoustic pitch matching methods, however, will remain a difficult endeavor, because there is currently no outcome measure that can be used for validation.

Although the binary search procedure does not require *a priori* assumptions on the frequency range of the acoustic stimuli, in the present study, this was not strictly the case since the acoustic frequency range was purposely limited to values ranging from 125 Hz to 4 kHz. The maximum frequency limit of 4 kHz was imposed because a pilot experiment with normal-hearing (NH) subjects showed that when using a 4-kHz standard, pitch matching accuracy for NBN and PSHC was substantially decreased. In addition, we did not anticipate that stimulation of the middle electrode (E6), which was the most basal electrode tested, would elicit pitch sensations higher than 4 kHz. Based on the results from the previous study using sinusoids (Rader et al., 2016) and on CF estimations using X-ray images, we expected E6 to be matched in the range 1–2 kHz, which was generally true for our results using SINE (see Figure 5 and Table 2).

### Methodological Considerations

The present study involved several methodological features with respect to the loudness-balancing procedure, the preliminary unilateral pitch comparisons, and the addition of catch trials to the main pitch matching task.

First, electric-acoustic pitch matching studies require that the acoustic and electric stimuli are equated in loudness before the subjects compare them in pitch. In most previous studies, the procedure was to first perform a *rough* pitch match between the acoustic and electric stimuli and then conduct loudness comparisons for these approximately pitch-matched stimuli (e.g., Schatzer et al., 2014). This approach may introduce an additional bias in that the subject could learn to associate a given electrode to a certain acoustic stimulus before starting the main pitch matching experiment. To avoid this potential problem, the electrodes used in this study for the loudness balancing (E3 and E4) were different than those used for the pitch matching (E1 and E6). In an earlier study, Vermeire et al. (2008) also limited the amount of loudness comparisons between the electrodes and the acoustic stimuli by only balancing one middle electrode (E6) to the acoustic sounds and then balancing all other electrodes to this middle electrode.

Second, prior to collecting electric-acoustic pitch data, each subject performed unilateral pitch comparisons separately in each modality. The electric-electric comparisons were used to verify that the two electrodes used in the main procedure (E1 and E6) were tonotopically ordered in the ‘electric’ pitch dimension such that subjects did not show pitch reversals. Despite the relatively large distance between neighboring electrodes of the MED-EL Flex28 electrode array (2.1 mm), only 4 out of 13 subjects could perfectly pitch rank electrodes E1 to E8. Furthermore, the variability in the ranks was larger for the 4 apical than for the 4 basal electrodes tested, consistent with previous studies in subjects with deep electrode insertions (Gani et al., 2007; Kenway et al., 2015). This suggests that the place pitch percept produced by different electrodes may not be very salient and could further depend on relative changes in the quality of sound, or timbre, as shown in a previous study using multi-dimensional scaling (Vermeire et al., 2013). The acoustic-acoustic pitch matching allowed the subjects to get accustomed to the procedure and was also used to compare their ability to match the pitch of acoustic sounds to that of NH listeners, which was largely comparable between groups for the 250-Hz and 1-kHz standards.

### Effect of Acoustic Stimulus Type

Comparing the pitch of sounds with different timbres is known to be a difficult task (Micheyl and Oxenham, 2004; Carlyon et al., 2010). Carlyon et al. (2010) presented results from NH subjects tested with two procedures that used sinusoids in one ear and noise bands in the other ear and showed that, while the subjects could correctly pitch rank each type of sounds separately, their pitch match across ears were strongly influenced by range biases. It therefore appears essential to perform pitch comparisons between sounds that are relatively similar.

In the present study, we have investigated two types of broadband stimuli (NBN and PSHC), which were 1/3-octave wide and had 36-dB per octave spectral slopes. These slopes are broadly consistent with previous vocoder studies that aimed to simulate the sound of CI (reviewed in Mesnildrey and Macherey, 2015; Karoui et al., 2019). However, it is likely that the spectral slope corresponding to that of the excitation spread of a CI electrode will vary across electrodes and across subjects. In order to evaluate the impact of such variations, NH subjects (*n* = 10) were tested in a pilot pitch matching test. For each stimulus type (NBN or PSHC), they were asked to match a standard stimulus presented in one ear at a fixed center frequency (250 Hz, 1, or 4 kHz) with a comparison stimulus presented to the other ear with different spectral slopes (24, 36, or 48 dB per octave) and adaptively changed in frequency using the binary search procedure. For each stimulus type and standard frequency, results showed no significant differences between comparison spectral slopes. Therefore, we assume that the specific spectral slope used in the present study (i.e., 36 dB per octave) did not have a significant effect on the collected data.

The underlying hypothesis of our study was that PSHC would be perceptually closer to the sound of a CI electrode than NBN or SINE, and that pitch matches would consequently be less variable. This hypothesis could not be supported by the present data, which may be due to several reasons; first, the rate of PSHCs is defined based on the outputs of Gammatone filters which may not be valid for all SSD subjects who sometimes show hearing loss at high frequencies (Hilkhuysen and Macherey, 2014). Second, PSHCs do not simulate pulse-to-pulse interactions within a given pulse train, which are present in electrical hearing (Boulet et al., 2015). Third, PSHC can produce distortion products, particularly at a frequency corresponding to their rate (Hilkhuysen and Macherey, 2014), which are absent in direct electric stimulation of the auditory nerve via a CI electrode. The significantly higher across-subject pitch matches for PSHC compared with SINE may relate to this third point, because the temporal cue provided by the PSHC rate together with distortion products may have provided an additional pitch cue lower than the ‘spectral’ pitch cue corresponding to the center frequency of the stimulus. Therefore, it cannot be excluded that subjects adjusted the center frequency of the PSHC at a frequency higher than that of SINE to *compensate* for this additional pitch cue. However, the similarity between mean pitch matches obtained for NBN and PSHC is inconsistent with this explanation since this additional pitch cue is not present in NBN.

The finding that pitch matches corresponding to a given electrode depended on the type of acoustic stimulus warrants some caution when interpreting the results of electric-acoustic pitch matching studies because there is currently no scientifically based justification for using one type of sound over another. In the present study, for example, if one only considered pitch matches using SINE (see Figure 5 and Table 2), then one would conclude that the subjects were adapted to their speech processor’s center frequency at E6 of approximately 1.3 kHz despite the tonotopic mismatch (Reiss et al., 2007; Reiss et al., 2014) but not for E1. This conclusion, however, is not supported by the pitch matches using NBN or PSHC. Another recent study reported an effect of acoustic stimulus type on electric-acoustic pitch matching; Maarefvand et al. (2017) have used sinusoids and harmonic complex tones consisting of the first 11 harmonics passed through a bandpass filter with relatively shallow slopes and centered at a frequency equal to 1.6 times the fundamental frequency. The spectral shape of this stimulus was chosen based on the results of a study on timbre by Lazard et al. (2012). They found that the pitch-matched frequency was always higher for sinusoids compared with the complex tone. It is worth noting, however, that both the spectral centroid and the fundamental frequency of these complex tones co-varied, which may have made the comparison with electric pulses difficult since – presumably – only the place of excitation varied for the electric stimuli.

Goupell et al. (2019) raised another concern when comparing pitch between ears and modalities; they showed that range biases were strongly present in bilateral CI users when comparing the pitch of electrodes in different ears, although these same subjects could reliably pitch rank electrodes in each ear. They further suggested that differences in stimulation modalities (acoustic vs. electric) may not be the only problem associated with electric-acoustic pitch matching but that other yet unknown processes may make inter-aural pitch comparisons difficult.

### Effect of Electrode Position

The tonotopic organization of the cochlea is one of the main prerequisites for the functioning of a CI. Speech processing strategies utilize this physiological property by presenting different frequency information to discrete locations along the length of the electrode array (Macherey and Carlyon, 2014). However, different CI electrode arrays generally do not reach the apex of the cochlea and thus a mismatch between frequency allocations of the speech processors and the physiological tonotopy of the auditory nerve is presently inevitable (Landsberger et al., 2015). A histological study by Stakhovskaya et al. (2007) provided relative location maps for the organ of Corti (OC) and the spiral ganglion (SG). Using the angle of insertion for each electrode position, which is estimated from postoperative X-ray images, these maps are used to calculate place-dependent characteristic frequencies (CFs) according to the empirically modeled Greenwood function (Greenwood, 1990). Recent studies investigated manipulations of these frequency maps to improve pitch perception in CI users, but have thus far shown inconsistent results.

While this individualized approach could partly account for morphological variations of the cochlea (Rask-Andersen et al., 2012; Pietsch et al., 2017), some limitations need to be taken into account; first, there is an inherent error margin in estimations based on X-ray images due to, for example, a poor resolution of electrode contacts, or if the round window is not easily identifiable since it provides the 0° reference for the frequency maps (Stakhovskaya et al., 2007; Verbist et al., 2010; Koch et al., 2017). Second, some studies based their assumptions or compared their results with the SG map, which is presumed to be the locus of neural excitation via CI (Landsberger et al., 2015; Peters et al., 2016), while other studies including the present one found their results to be better approximated by the OC map, especially when using lateral-wall electrode designs (Vermeire et al., 2008; Schatzer et al., 2014). These discrepancies underline the fact that these models are based on assumptions which cannot always be held true and should be reconsidered depending on the study approach. Third, there is a growing body of literature regarding peripheral degeneration or dead regions of spiral ganglion cells and their effect on CI function (Pfingst et al., 2015), which could further have an effect on variability between subjects.

The present study showed an effect of discrete electrode position independent of acoustic stimulus type, which is in line with an underlying tonotopic organization. However, SG and OC maps showed different results depending on stimulus type (see Figure 7) such that no consistent conclusions could be drawn. These results together with the aforementioned limitations raise the question whether relative distances rather than absolute place-dependent frequency estimations are more important for pitch comparisons between electric and acoustic stimulation. As mentioned above, Vermeire et al. (2013) conducted a multi-dimensional scaling study and suggested that a change in place of stimulation, i.e., electrode position, results in a perceptual change concurrent with acoustic frequency. Still, they also found another dimension showing concurrent change, which can be attributed to a change in sound quality or timbre. It is assumed that this dimension can be dependent on other factors, such as neural survival or pulse-to-pulse interactions (Boulet et al., 2015; Pfingst et al., 2015), which is in turn dependent on the electrode position *per se*, but not necessarily based on empirical models. For example, Carlyon et al. (2010) tested a subject at two different time points who showed a substantial change in matched frequency, which was later found out to be consistent with electrode migration.

Since mean pitch matches were better predicted by CFs according to the OC map for all stimulus types, we can assume it as a model of place-dependent variations in our subject group. Given this premise, we speculate that the present data may shed light on the type of acoustic stimulus that best mimics the sound of a CI electrode. For the apical position (E1), SINE pitch matches were significantly closer to predicted CFs than either NBN or PSHC. And for the more basal position (E6), NBN and PSHC were closer to predicted CFs than SINE. This supports the notion that a change in place of stimulation could lead to a change in sound quality, with pure tones better mimicking the percept elicited by electric stimulation in the apical region and wideband stimuli in the basal region. Thereby providing a possible explanation for the high variability observed in previous studies attempting to match electric with acoustic pitch in SSD subjects, since the choice of acoustic stimulus may have been optimal only for a part of their data.

## Conclusion

Different acoustic stimulus types were used to match the pitch of electric stimulation with the underlying assumption that a signal perceptually more similar to how electric stimulation sounds should yield less variable pitch matches. While it was not possible to reject the null hypothesis regarding this effect, our data provide evidence that the choice of acoustic stimulus type can have a significant effect on electric-acoustic pitch matching results. Our results further confirm a stimulus-independent effect of electrode position which, however, is not necessarily predicted by absolute frequency mapping. This suggests that changes in place of electric stimulation are attributed to relative changes in pitch or timbre. Place-dependent characteristic frequencies predicted by an organ of Corti map suggest sinusoidal sound quality in the apex and one closer to wideband stimuli in the base. Further research is still needed to find an acoustic stimulus that better mimics the percept elicited by electric stimulation in order to investigate how cochlear implant users process pitch information.

## Data Availability Statement

The raw data supporting the conclusions of this manuscript will be made available by the authors, without undue reservation, to any qualified researcher.

## Ethics Statement

This study was carried out in accordance with the ethical standards of the institutional review board at the Goethe University Frankfurt, which approved the study protocol (IRB approval number 209/13). All subjects gave written informed consent in accordance with the Declaration of Helsinki.

## Author Contributions

YA, TW, UB, and OM contributed to the conception and design of the study. YA and SN organized and conducted the experiments. YA and OM performed the statistical analysis. YA, TW, UB, and OM contributed to the interpretation of the data. YA and OM wrote the first draft of the manuscript. All authors contributed to manuscript revision, read and approved the submitted version.

## Conflict of Interest

The authors declare that the research was conducted in the absence of any commercial or financial relationships that could be construed as a potential conflict of interest. The reviewer JK declared a shared affiliation, with no collaboration, with several of the authors, YA, SN, TW, and UB, to the handling editor at the time of review.
